# Comment on “Assessment of Ambient Air Pollution in Istanbul during 2003–2013”

**Published:** 2018-11

**Authors:** Marzieh Mahmoudimanesh

**Affiliations:** Dept. of Biostatistics & Epidemiology, School of Public Health, Kerman University of Medical Sciences, Kerman, Iran

## Dear Editor-in-Chief

In the previous work by Eray Yurtseven et al (1), helpful information was provided on air pollution in Istanbul during the past few years, but there were some problems with the paper.

In a table of this paper, the minimum values of CO were not in accordance with its maximum values. For instance, the minimum value in 2003 was stated as 280, while a smaller value, i.e. 3.963, was mentioned as the maximum value.

It was stated in that paper that the European and Asian parts were significantly different in terms of pollution, but no population adjustment had been carried out. Since population can be an important factor in regard to pollution, the results should have been stated given population and its adjustment (2).

The study can actually be regarded as a kind of descriptive research, and time series methods could preferably have been utilized for further analysis so that conclusions could have been made through adjustment of different factors effective on pollution. The results would probably have changed in that case.
